# Design and optimization of an innovative lining structure for high-pressure water transmission tunnels subjected to strike-slip fault creep

**DOI:** 10.1038/s41598-025-88602-3

**Published:** 2025-03-27

**Authors:** Wen-Tao Xu, He-Gao Wu, Chang-Zheng Shi, Yong Xia, Xing-Yi Yang

**Affiliations:** 1 State Key Laboratory of Water Resources Engineering and Management, Wuhan University, Wuhan, 430072 Hubei People’s Republic of China; 2 Three Gorges New Waterway (Hubei) Co., Ltd., Yichang, 443000 China; 3 Chengdu Engineering Co., Ltd., Power China, Chengdu, 610000 China

**Keywords:** High-pressure tunnel, Lining structure, Fault creep, Numerical simulation, Optimization analysis, Civil engineering, Hydroelectricity

## Abstract

Long-distance water transmission tunnel projects face significant challenges in crossing active faults. This paper presents a novel multi-layer flexible lining (MFL) structure for high-pressure tunnels that can accommodate fault creep deformation. Finite element numerical analysis was utilized to validate the feasibility of the MFL structure under the actual conditions of a particular engineering project. Additionally, the effects of various parameters on the tunnel structure were examined. The results indicated that shorter concrete segments and longer flexible joints are better able to accommodate fault dislocations and reduce concrete damage. A thicker cushion layer, with a thickness ranging from 0.1 to 0.4 m, is more advantageous for the tunnel lining to adapt to fault dislocations. However, an excessively thick cushion layer will have a negative impact on the lining’s stress. Appropriate use of bellows joints can improve the tunnel resistance to fault displacement. The number of bellows joints needed for effectiveness and the potential failure of the joints at the edge of the fault zone were examined. The research findings can provide valuable guidance for the structural design of high-pressure water transmission tunnels in dealing with fault creep deformation.

## Introduction

The incongruity between the spatial distribution of water resources and regional social development has become an increasingly pressing global issue for economic development^1^. In an effort to address this issue, numerous nations have undertaken the design and implementation of a multitude of inter-basin water transmission projects in recent decades, such as the South-to-North Water Diversion Project in China^2^, the Central Arizona Project in the USA^3^, the Gerede Tunnel Project in Turkey^4^, and the Lesotho Highlands Water Project in Southern Africa^5^. These projects often involve the construction of long-distance tunnels, many of which are situated in mountainous areas with complex geological formations and high seismic activity^6,7^. Consequently, crossing active faults is a common challenge during tunnel construction.

**Fig. 1:**
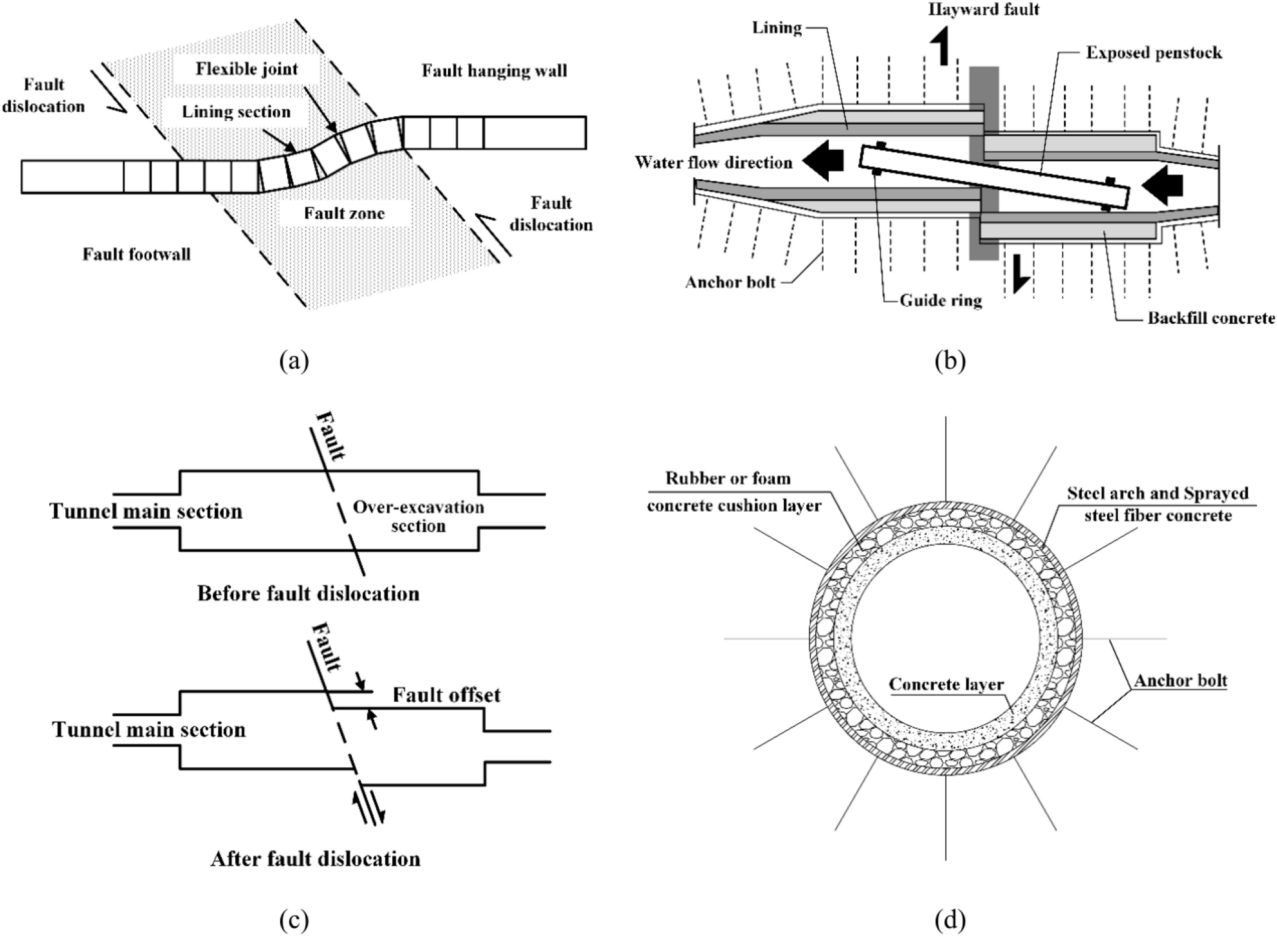
Schematic diagrams for anti-dislocation measures of tunnels: (**a**) use of articulated design, (**b**) exposed pipe crossing method within tunnel, (**c**) cross-section over-excavation method, and (**d**) use of energy dissipation or strengthening design with composite lining.

Tunnels that traverse active fault zones are vulnerable to both seismic shaking and fault dislocations, but it is widely believed that fault dislocations are the primary factor responsible for tunnel damage^8,9^. At present, anti-dislocation measures for tunnels typically include (a) the use of an articulated design, (b) the exposed pipe crossing method within the tunnel, (c) the cross-section over-excavation method, and (d) the use of an energy dissipation or strengthening design with a composite lining. These measures are schematically represented in Fig. 1. The schematic representations throughout this paper, including Fig. 1, were created using VISIO 2016 software.

In recent years, scholars from various countries have conducted extensive research on the failure mechanism and measures to resist the fault dislocations of tunnels passing through active faults. Among the various anti-dislocation techniques, the articulated design technique has been utilized for the tunnel construction of the Bolu tunnel in Turkey and the Koohrang-III water transmission tunnel in central Iran^10,11^. This method divides the tunnel lining into sections and installs flexible connection segments, such as shear joints or deformation joints, between each lining section to avoid overall damage to the lining. A shorter segment length is preferred to resist failure due to fault rupture^9,12^.

The cross-section over-excavation method, which is also commonly used in highway and railway tunnels, is another effective measure to resist fault dislocations. This method involves excavating the cross sections of tunnels adjacent to the fault when fault displacement is concentrated in specific areas. This ensures that the tunnels still have sufficient space after displacement^13,14^. Although an oversized tunnel can provide a sufficient cross section, it may not prevent the possibility of faulted gravel falling into the tunnel and contaminating the water supply. To prevent tunnel blockages or water pollution caused by tunnel crown collapse, an internal carrier pipe can be installed within the oversized tunnel section, which can ensure the normal operation of the tunnel following fault movement^15^. In addition, various new measures to resist fault dislocations, including the use of novel structures with multiple layers and concrete with special properties, have been continuously proposed^16–21^. The above methods can be summarized into two types based on the design philosophy of the reinforced concrete lining: (1) changing the performance of the underground structure itself to adapt to fault deformation, such as by using an articulated design and an energy dissipation or strengthening design with a composite lining, and (2) ensuring structural function after fault dislocation, such as by using the cross-section over-excavation method and the exposed pipe crossing method within the tunnel. The former method is less capable of resisting axial tensile deformation, which reduces the ability of the lining to bear the surrounding rock load and cannot be used for high internal pressures. However, the latter method is only applicable to projects with smaller tunnel section sizes, and enlarging the excavation size requires a higher support cost to ensure the stability of the surrounding rock.

Tunnel engineering commonly employs reinforced concrete linings. When passing through fault zones with poor surrounding rock conditions, the reinforced concrete lining is prone to cracking and water leakage. Therefore, steel linings are often used for tunnels with high internal pressures, as they offer high strength and tightness, ensuring safe operation under normal conditions^22^. Nonetheless, conventional steel-lined tunnels exhibit poor shear and compressive resistances. Active fault movement can cause S-shaped bending deformation and buckling failure of steel linings, especially under the extrusion effect of a reverse fault. Anti-shear and anti-compressive improvements are still necessary for the use of steel linings in high-pressure tunnels crossing active faults. Unfortunately, there has been little research in this area.

In this study, a multi-layer flexible lining (MFL) was proposed for high-pressure water transmission tunnels crossing active faults to address the issues of anti-dislocation. Finite element models of different tunnel lining structures were established based on an actual project. The primary objective was to validate the rationality of the MFL by comparing the stress and deformation levels when other structures and the MFL structure were subjected to strike–slip fault creep. Subsequently, numerical simulations were conducted to analyze the influences of various factors on the internal force response and fracture resistance of the MFL structure, providing directions for structural optimization. Moreover, guidance for the design of water transmission tunnels with high water pressures is provided based on the findings of this study.

## Conventional and innovative structures

**Fig. 2:**
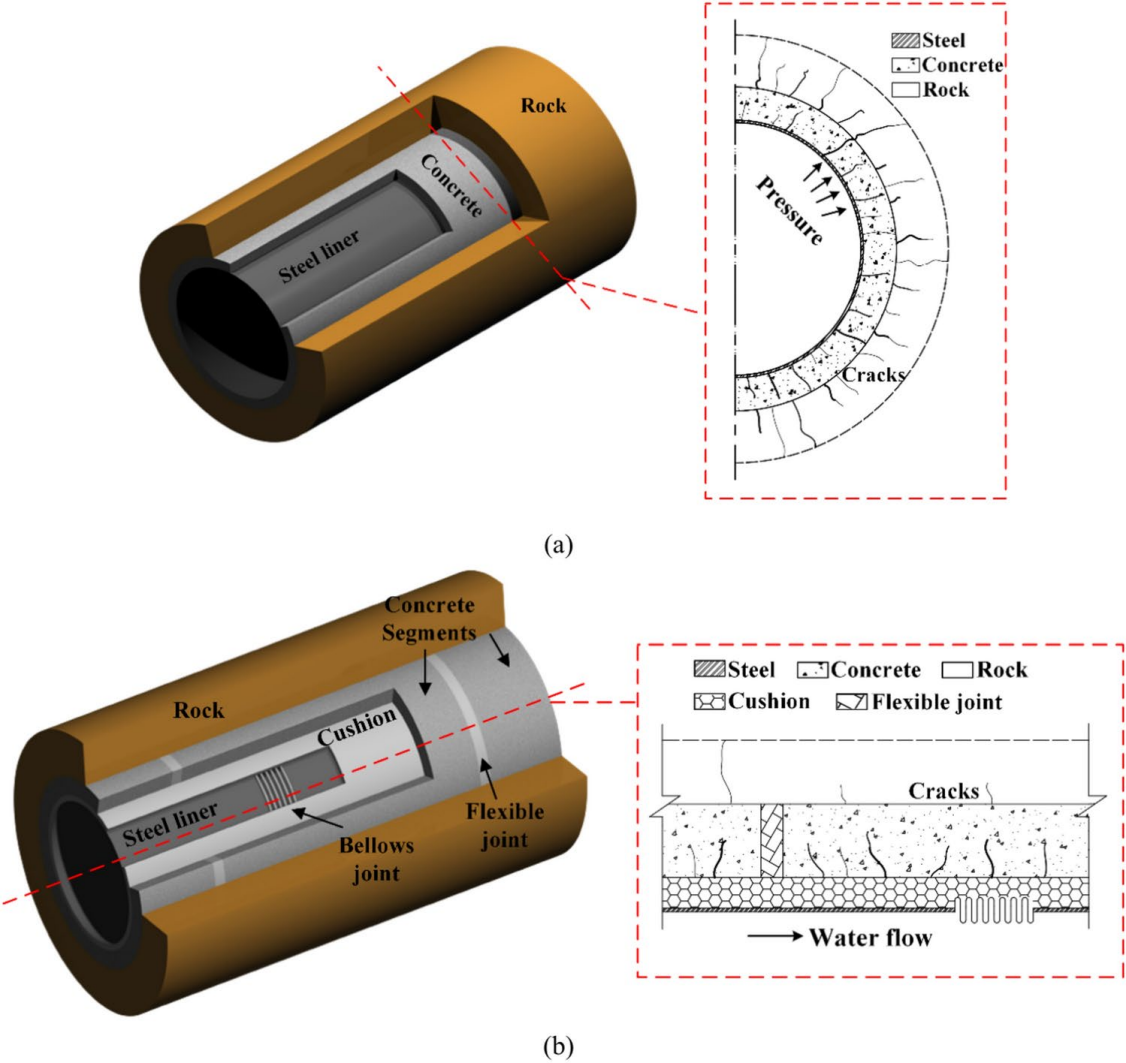
Schematic diagrams of the two types of tunnel lining structures: (**a**) steel-lined tunnel and (**b**) multi-layer flexible lining (MFL) tunnel.

To illustrate the differentiation between the steel-lined structure and the MFL structure, sketches of both tunnel lining structures are presented in Fig. 2. A steel lining is commonly used in tunnel sections where the minimum principal stress of the surrounding rock is less than the internal tunnel pressure^23^. The most widely used type of this lining is the steel pipe external backfill concrete. The use of steel as a lining material provides excellent structural airtightness and durability, while the external backfill concrete helps to distribute loads evenly and avoid damage to the steel lining. It allows the concrete to have some small radial cracks in operation, as illustrated in Fig. 2a.

In response to the issues encountered with steel-lined structures across faults, the MFL structure is proposed, as illustrated in Fig. 2b. The structure consists of a backfill concrete layer, cushion layer, and steel liner from outside to inside. The intermediate cushion layer is designed to provide the steel liner with a certain amount of deformation space and absorb some of the displacement transmitted from the concrete. It creates a structural displacement discontinuity area. The flexible joints, made of either foam concrete or fiber-reinforced plastic concrete, are installed at regular intervals in the backfill concrete, with a set elastic modulus of 1/200 of the elastic modulus of the concrete in the lining segment, while the other parameters remain unchanged^24^. The flexible connection segment has a smaller elastic modulus and can concentrate the shear and compressive forces of the fault on itself more effectively than the concrete can, thus avoiding overall damage to the concrete. Multiple bellows joints are added to the steel liner to improve its flexibility and adaptability to the fault’s displacement within the protective distance.

## Project overview and calculation model

### Basic information

**Fig. 3:**
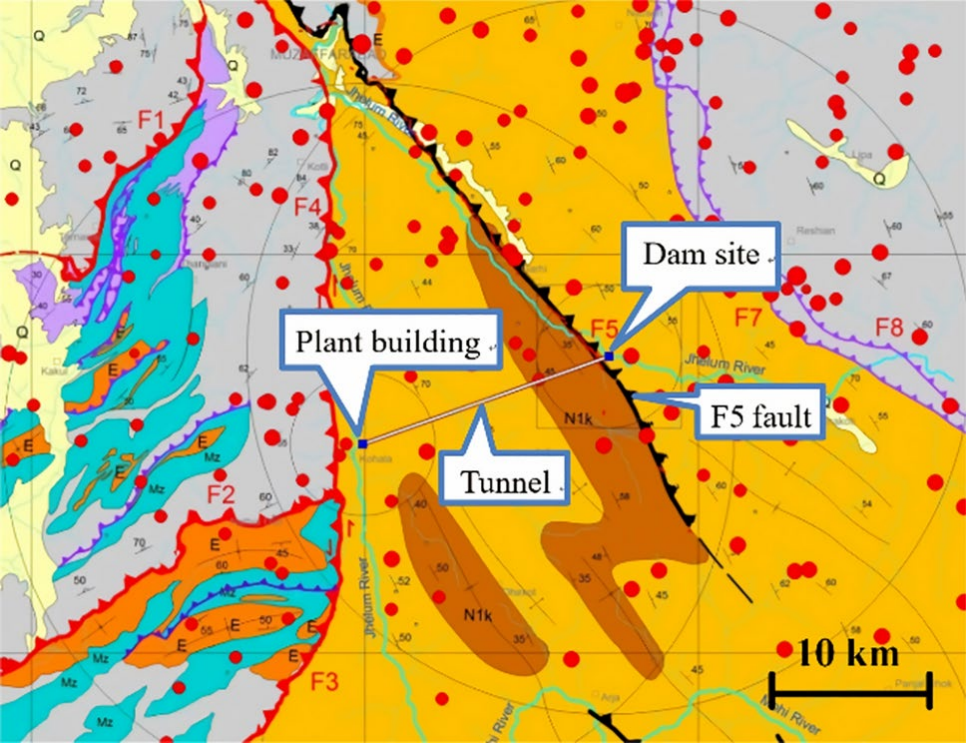
Schematic diagram of the extension of active fault zone.

A hydropower station in the northeast of Pakistan was selected as the research subject. The diameter of the diversion tunnel is 5 m, and the length is about 17.4 km. The design internal pressure is 1.5 MPa. A steel lining is used in the area approximately 700 m before and after the fault zone, while reinforced concrete linings are used in other areas. The active fault zone that intersects with the diversion tunnel is the reverse fault F5, as illustrated in Fig. 3.

The main dam site of the hydropower station is located on the hanging wall of the active fault. The F5 fault passes through the upstream section of the diversion tunnel, and the overall dip angle of the F5 fault zone is about 55°–60°. The surrounding rock at the dam foundation is affected by the reverse fault of the F5 fault zone and is relatively fragmented. Based on the Q system classification method^25^, the surrounding rock categories of the intersection section between the fault zone and the tunnel are mainly class Q5, followed by class Q6, The physical and mechanical properties of the rock mass used in the three-dimensional (3D) numerical model, as listed in Table 1, were obtained from the multiple exploratory boreholes in this region.

**Table 1:** Material parameters of the surrounding rock.

Name	Rock category	
	Q6	Q5
Bulk density (g/cm^3^)	2.15–2.19	2.43–2.45
Friction angle (°)	22–30	34–41
Poisson’s ratio	0.34–0.38	0.31–0.34
Elastic modulus (GPa)	0.5–1.5	4–8
Deformation modulus (GPa)	0.3–1.1	2–4
Cohesion (MPa)	0.14–0.26	0.41–0.82

The steel lining is made of Q355 steel with a yield strength of 345 MPa. The material parameters of the cushion and concrete are summarized in Table 2. The friction coefficients between the materials are shown in Table 3. It should be noted that the material parameters presented in Tables 2 and 3 were mostly obtained through rigorous experimental testing, and a small portion were derived from relevant publications^24–26^ after ensuring the reliability of their sources.

**Table 2:** Material parameters of the cushion and concrete.

Materials	Bulk density (kg/m^3^)	Poisson’s ratio	Elastic modulus (MPa)	Axial compressive/tensile strength design value (MPa)
Concrete	2400	0.167	2.55 × 10^4^	9.6/1.1
Flexible joint	2400	0.167	128	–
Cushion	500	0.16	3	–

**Table 3:** Friction coefficients for MFL.

Material	Friction coefficient
Surrounding rock and concrete	0.86
Concrete and flexible joint	0.70
Cushion layer and concrete	0.63
Steel liner and concrete	0.47
Steel liner and cushion layer	0.30

### Finite element model

**Fig. 4:**
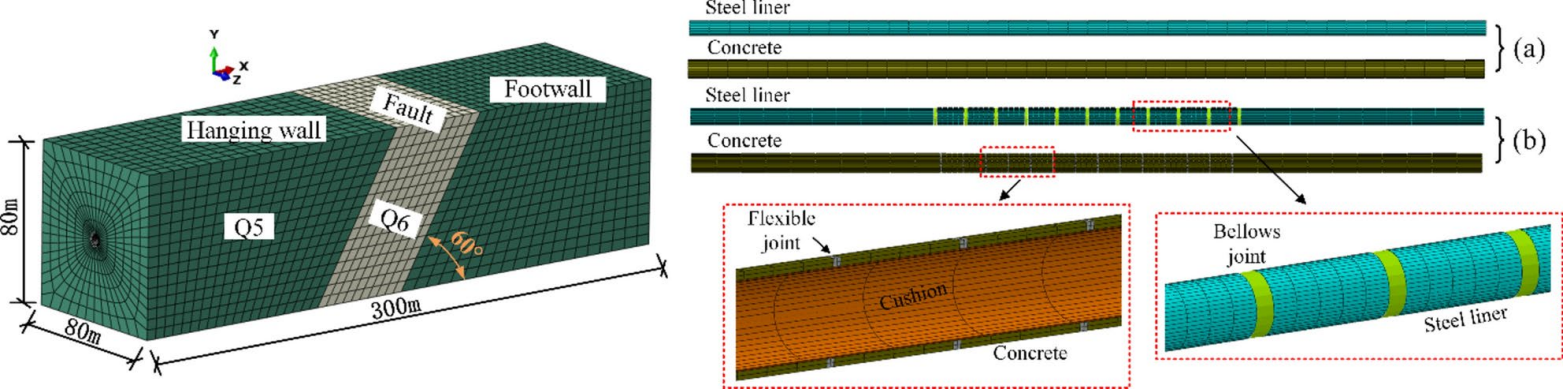
Finite element models: (**a**) steel-lined tunnel and (**b**) MFL tunnel.

Based on the above project data, numerical models of the water transmission tunnel using different lining types were established, as shown in Fig. 4. Some key structural parameters are shown in Table 4. In the MFL structure, the flexible joints and bellows joints were uniformly distributed in the engineering fortification zone. In order to avoid the influence of the boundary conditions on the finite element calculation results^27^, the cross-sectional dimension of the model was selected as 80 m × 80 m, and the longitudinal (along the pipe axis) length of the model was 300 m. The longitudinal length of the fault zone was 50 m, and the fault dip angle was determined to be 60°.

**Table 4:** Structural parameters and dimensions for MFL.

Parameter	Length (m)	Parameter	Radius/thickness (m)
Flexible joints	0.5	Steel pipe radius	2.5
Concrete segments	8	Backfill concrete thickness	0.5
Steel pipe segments	10	Steel wall thickness	0.024
Bellows joints	1.5	Cushion layer thickness	0.1

In the modeled system, the steel liner was represented by the four-node S4R shell element, whereas the lining concrete, surrounding rock, cushion layer, and other materials were represented by eight-node C3D8R solid elements utilizing the ABAQUS 2016 software. The axial and transverse stiffness values of the bellows joint, 1274 and 1481 N/mm respectively, were obtained through manufacturer testing. Processing the bellows joint through three-dimensional shell elements was possible, but the bellows pipe, having a special geometry, would comprise a very large part of the finite element model, regardless of its small size. To avoid inefficiency, the bellows joint was simulated using the B31 beam element with a circular ring section. The axial modulus and transverse stiffness (these two parameters were required in ABAQUS) of the beam element were calculated based on the stiffness of the bellows joint^28,29^.

**Fig. 5:**
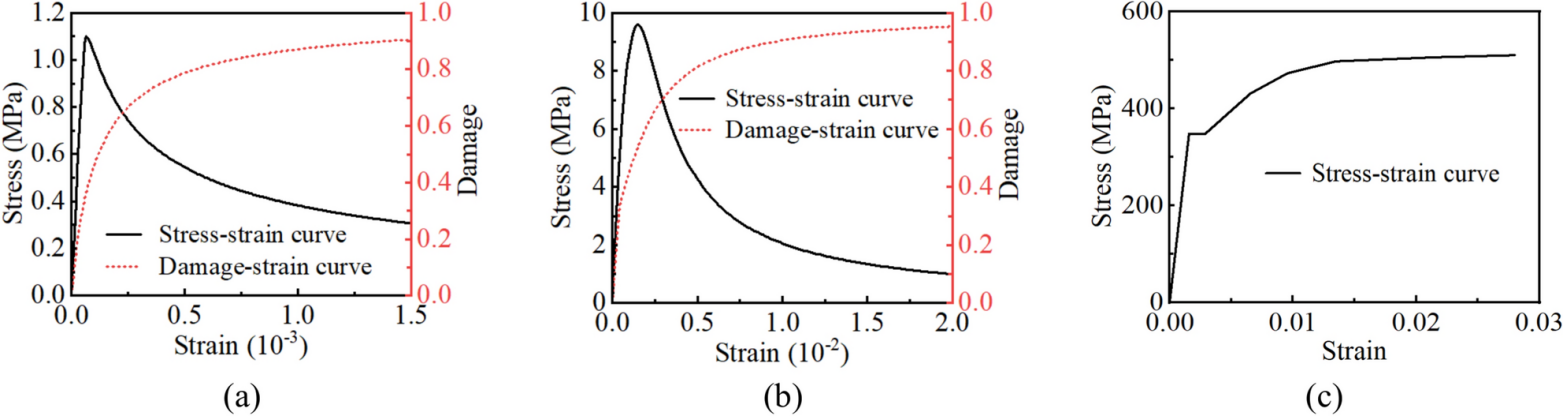
(**a**) Tensile stress–strain and damage curves of concrete, (**b**) compressive stress–strain and damage curves of concrete, and (**c**) stress–strain curve of steel.

The elastic constitutive model is commonly used for lining structures^30^, and the flexible joints and cushion layer were simulated using a linear elastic constitutive model in this numerical model. However, the internal structure of concrete is complex both in its macro- and micro-sizes. It is neither comprehensive nor reasonable to use the linear elastic constitutive model to describe the properties of concrete. The concrete lining of the tunnel was modeled using the concrete damaged plasticity model developed by Lubliner^31^. The steel liner was modeled using a multi-linear kinematic hardening constitutive model. Figure 5 shows the stress–strain curves for both materials. The Mohr–Coulomb constitutive model was used for the surrounding rock and fault zone. Due to the differences in the mechanical properties of each material, relative opening, closing, and sliding may occur between layers, so contact surfaces were set between the various materials, including the backfill concrete and surrounding rock, steel liner and concrete, and steel liner and cushion layer, to simulate the force transfer behavior of the interfaces. In this study, only the normal operating conditions were considered, and the influence of the surrounding rock pressure was ignored.

### Boundary conditions

**Fig. 6:**
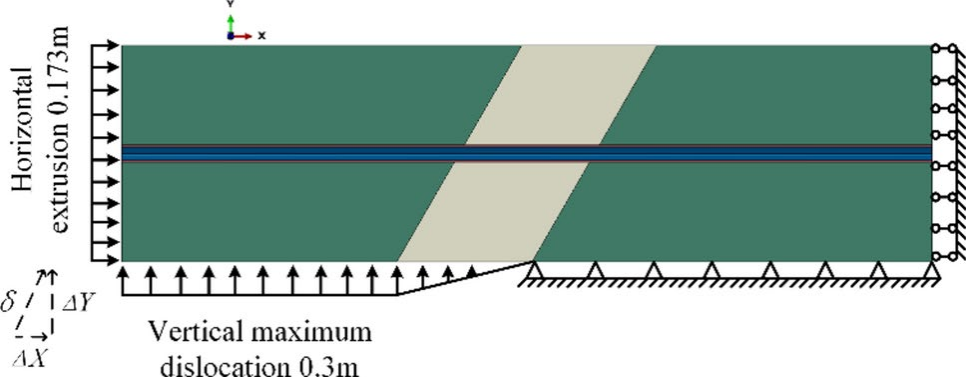
Boundary conditions.

The method of imposing a forced static displacement to the rock is usually adopted to simulate a fault creep dislocation. In accordance with the engineering fortification requirement, the relative creep displacement between the hanging wall and footwall of the fault was 0.35 m (δ). It was decomposed into two components. Specifically, the fault displacement in the positive Y-direction (Δ*Y*) of the hanging wall was 0.3 m, while the fault displacement in the positive X-direction (Δ*X*) was 0.173 m. It was a kind of ultimate limit boundary condition for the structure. The faulting displacement distribution patterns within the fault zone lacked verification from on-site measured data. However, there are four distribution patterns commonly used currently: convex-upward, linear, concave-downward, and S-shaped patterns^32,33^. The most commonly used linear pattern was adopted in this study, as shown in Fig. 6.

### Verification of numerical approach

**Fig. 7:**
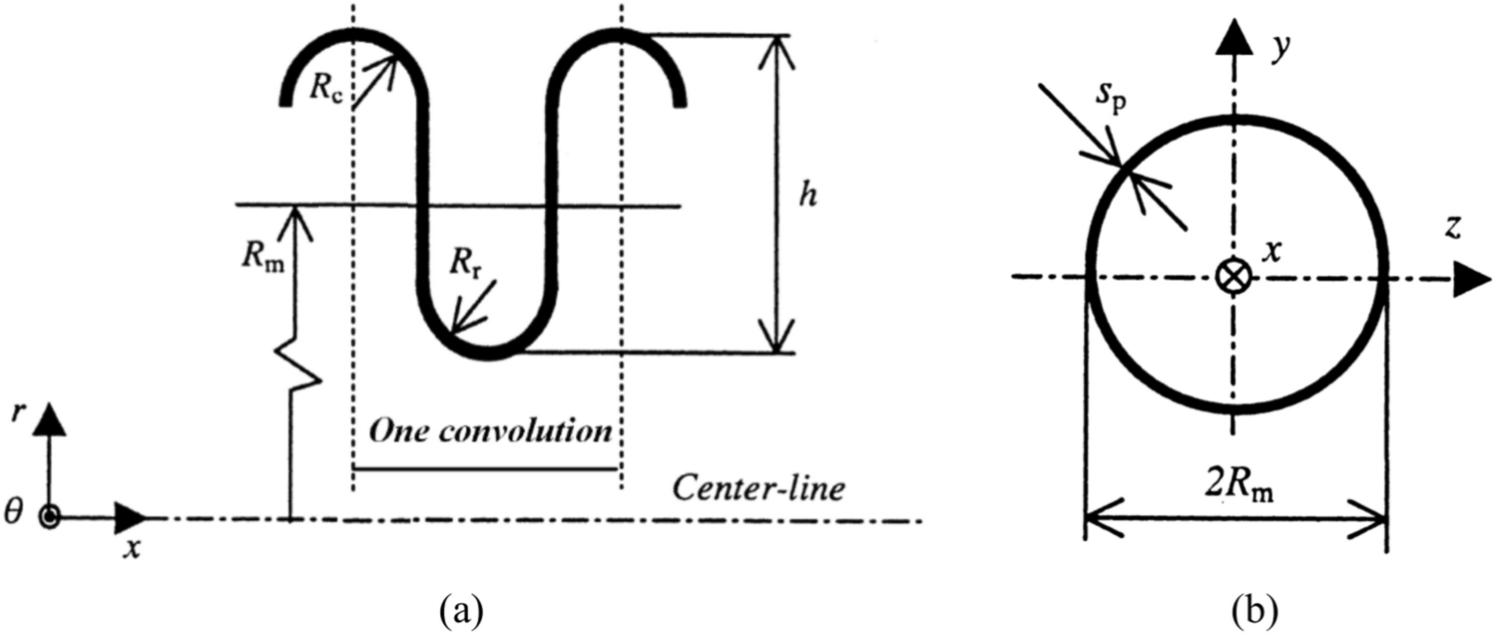
(**a**) U-shaped convolution geometry and (**b**) thin-walled pipe analogy of bellows joint.

Before our systematic parametric study on the influence of essential factors of MFL structures on their anti-fracture performance, we must first ensure the accuracy and reliability of our simulation method. Xiang^34^ carried out tests on four-convolution single bellows joints under low frequency cyclic loading. The thickness of the bellows joint was 4 mm, and the cyclic loads were controlled by cyclic displacements with increasing amplitudes of 2, 3, 4, and 5 mm. The formula proposed by Broman^28^ can be used to calculate the axial modulus and transverse stiffness of the beam element based on the relevant parameters of the bellows pipe in the physical experiment. The geometry of a U-shaped convolution is defined in Fig. 7a. By defining special values for the wall thickness and material properties, this pipe can be a good model of the corresponding bellows pipe, as shown in Fig. 7b. The equations for defining the materials properties, taking axial modulus of elasticity as an example, are presented as follows. The relation between the total axial stiffness, $k_{T}$, and the axial stiffness of one convolution, $k$, is

1 $$k_{T} = \frac{k}{n},$$

where $n$ is the number of convolutions. The axial stiffness of the equivalent pipe, $k_{p}$, is

2 $$k_{p} = \frac{{E_{p} A_{p} }}{L},$$

where $E_{p}$ is the modulus of elasticity of the pipe material,

3 $$L{ = }2(R_{r} + R_{c} )n,$$

and the cross-sectional area of the pipe is

4 $$A_{p} = 2\pi R_{m} s_{p} .$$

Setting $k_{p}$ equal to the total axial stiffness of the bellows joint yields

5 $$E_{{\text{p}}} = \frac{{k_{{\text{T}}} L}}{{A_{{\text{p}}} }} = \frac{{k_{{\text{T}}} L}}{{2\pi R_{{\text{m}}} s_{{\text{p}}} }} = \frac{{k(R_{{\text{r}}} + R_{{\text{c}}} )}}{{\pi R_{{\text{m}}} s_{{\text{p}}} }}.$$

With this modulus of elasticity, the equivalent pipe will have an axial stiffness equal to that of the corresponding bellows joint.

**Fig. 8:**
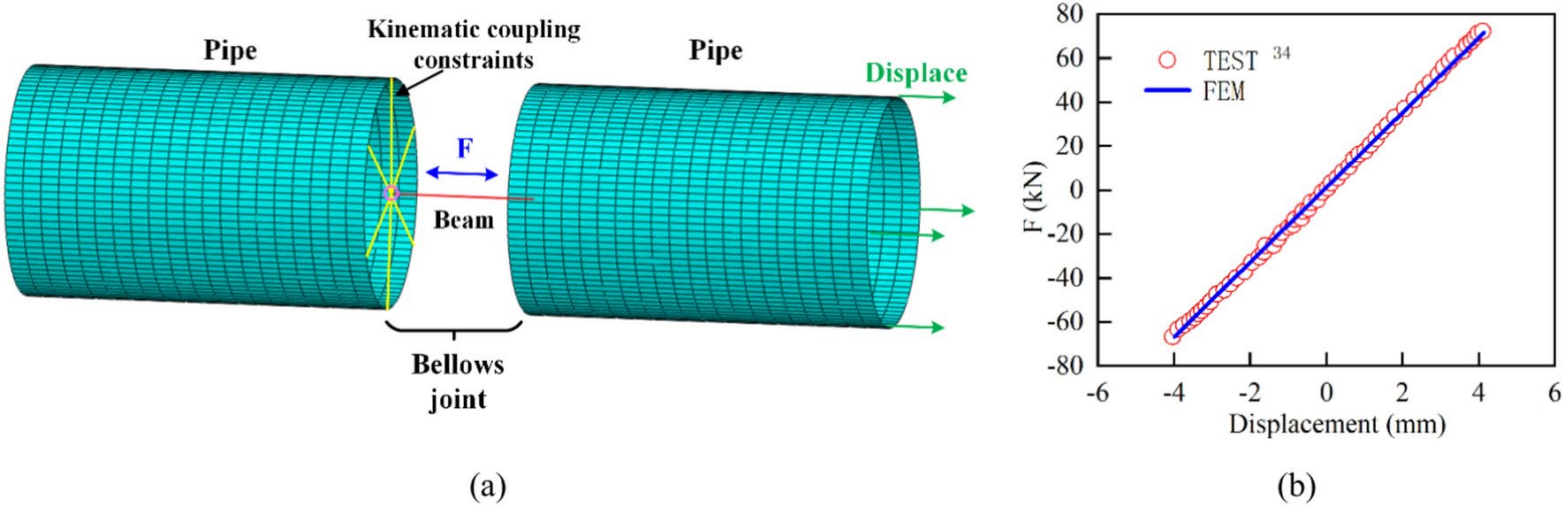
(**a**) Simplified model of bellows joint and (**b**) comparison of force–displacement curves from experiments and finite element results with 4-mm displacement amplitude.

Figure 8a presents the meshed simplified model of the bellows joint, where both sides of the beam element were connected to the shell element through kinematic coupling constraints. During the loading process of the experiments, bending deformation occurred when the displacement amplitude exceeded 5 mm. Figure 8b shows a force–displacement curve obtained from the simplified model with a displacement amplitude of 4 mm, which was compared with the results obtained during the experiment. From the figure, it can be seen that there was good agreement between the two. This consistency was also reflected in the force–displacement curve for transverse shear. Thus, it was demonstrated that this simplified modeling approach was effective.

**Fig. 9:**
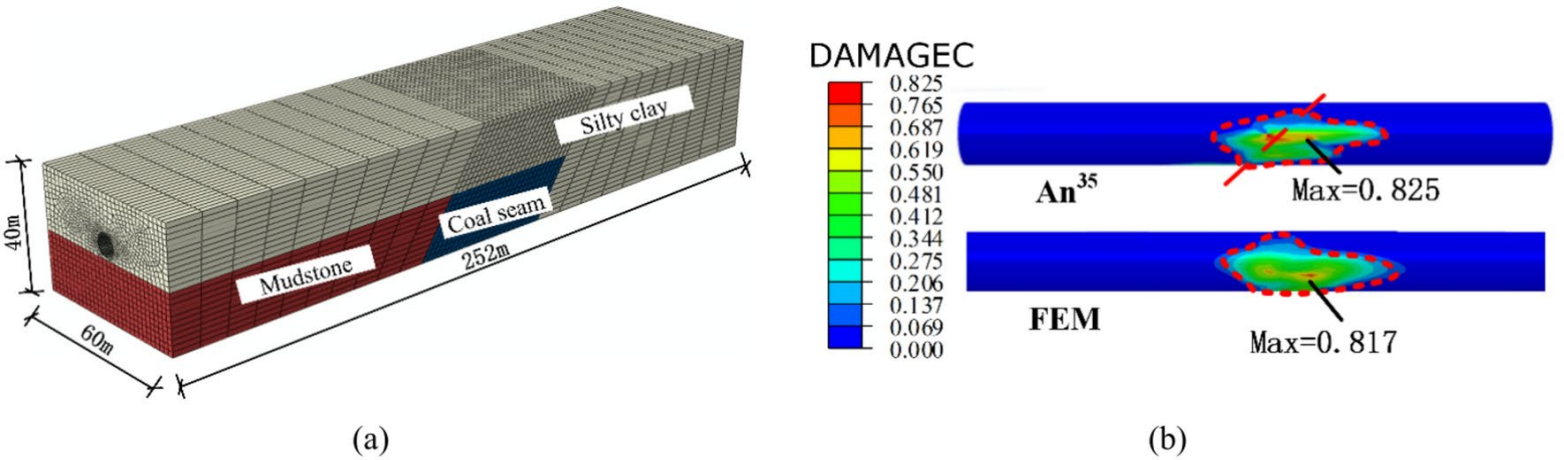
(**a**) Three-dimensional finite element model and (**b**) comparation of structural damage.

Concrete crushing is one of the most common forms of failure when large underground structures cross active faults. The Urumqi Metro tunnel mentioned in a previous publication was selected as an example, and a 3D model was established, as shown in Fig. 9a. The compression damage results after a fault displacement of 0.165 m were compared with An’s result^21^, as shown in Fig. 9b. It can be seen that the maximum damage value of the concrete tunnel calculated in the present simulation was similar to that in the literature, and the damage contours were also similar. Thus, the contact methods and damage parameters used in this article were reasonably established, and the subsequent calculation results have certain reference significance.

## Performance of steel-lined tunnel subjected to fault creep

**Fig. 10:**
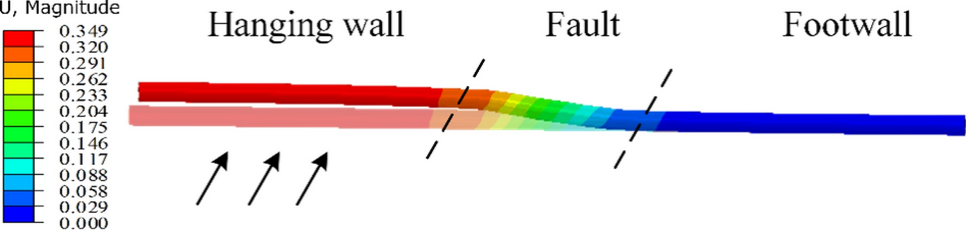
Resultant displacement of steel liner under fault conditions (m).

The primary focus of this section is to investigate the structural deformation of the conventional steel-lined tunnel under the action of fault movement. The tunnel lining structure utilized in this study is illustrated in Fig. 2a and consisted of a steel liner and backfill concrete. The lining underwent dislocation with the surrounding rock during the creep and slipping process of the fault zone, and the displacement of the steel pipe is shown in Fig. 10. This indicated that the deformation of the steel pipe was primarily concentrated in the fault zone. As the fault dislocation increased, the pipeline experienced increasing displacement and deflection.

**Fig. 11:**
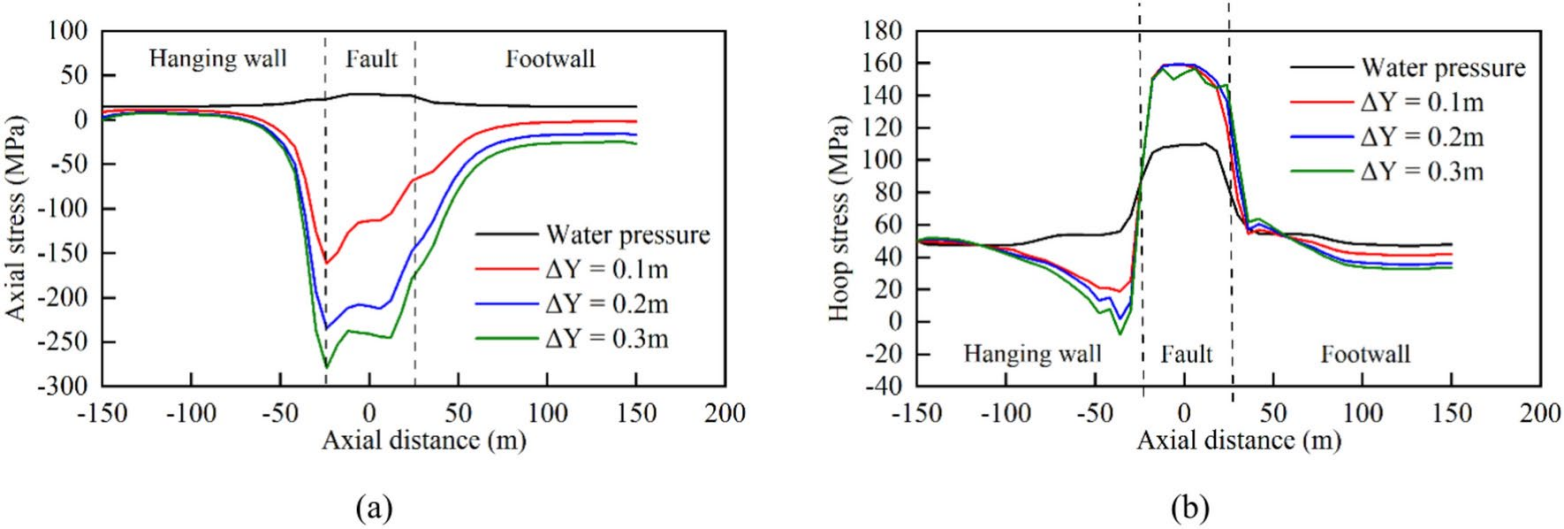
Stress curves of steel pipes under different fault displacement amounts: (**a**) axial stress and (**b**) hoop stress.

The tunnel was significantly influenced by the surrounding rock when displacement occurred in the hanging wall, due to the combined effects of gravity, water pressure, and fault extrusion. This led to increased stress concentration at the bottom of the steel pipe. The X-axis origin was set at the center of the fault zone. Curves depicting the stress variations along the pipeline axis are shown in Fig. 11.

The curves indicate that the hoop stress of the steel pipe during normal tunnel operation was mainly influenced by the water pressure and water weight. The small deformation modulus of the fault zone resulted in slight subsidence of the middle section of the pipe, causing slightly elevated stress in the steel pipe between the fault zones compared to the stresses on the left and right sides. The axial stress was primarily generated by the Poisson effect of the hoop stress due to the internal pressure, with most numerical values below 20 MPa. When the vertical dislocation of the fault (*ΔY*) was 0.1 m, the axial and hoop stresses of the steel pipe exhibited a large abrupt change near the left and right boundaries of the fault zone, and the change in the axial stress was greater than that of the hoop stress. With increasing fault dislocation, the steel pipe experienced continuous compression, leading to a gradual increase in the axial stress until it approached the yield point. Meanwhile, the hoop stress remained relatively stable overall.

**Fig. 12:**
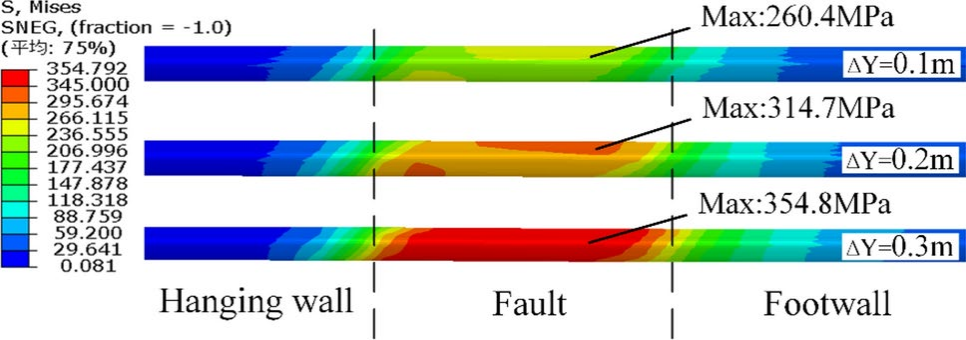
Stress distributions of steel pipes under different fault displacement amounts.

Under different amounts of fault displacement, the maximum value of the von Mises stress appeared at the intersection of the footwall with the fault zone, and the distribution map of the von Mises stress is shown in Fig. 12. When the vertical dislocation of the fault was 0.3 m, the von Mises stress within the fault zone of the steel pipe exceeded 345 MPa, resulting in a significant portion entering the plastic stage. The distribution of the von Mises stress was consistent with those of the axial and hoop stresses. The stress underwent significant changes within the fault zone and extended to 30 m (six times the diameter) on both sides. The range of stress impact was relatively insensitive to the amount of fault displacement.

**Fig. 13:**
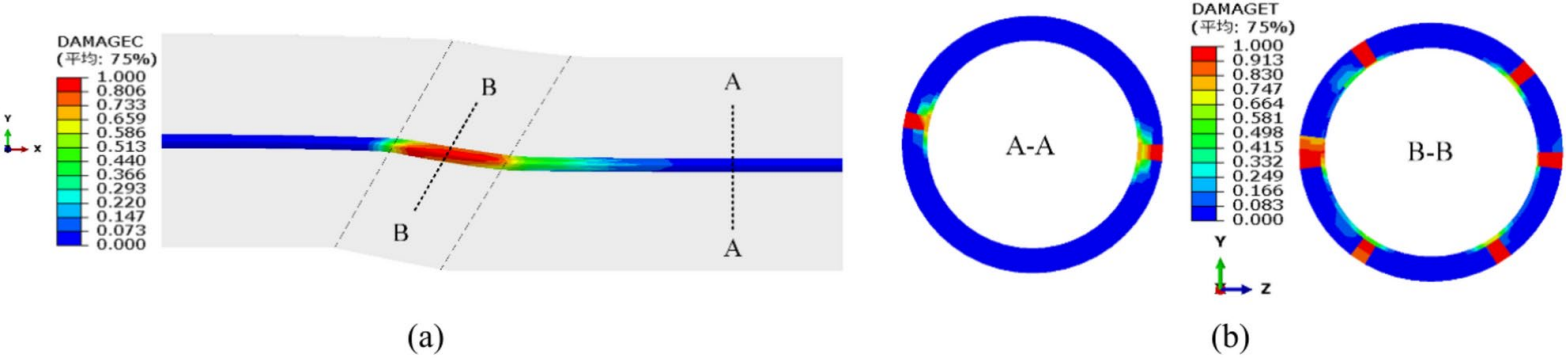
Damage of the concrete: (**a**) compressive damage and (**b**) tensile damage.

Apart from the large-scale deformation of the steel pipe, the backfill concrete also suffered from significant tensile and compressive damage, as shown in Fig. 13. The footwall area of the structure exhibited a larger range and severity of both compressive and tensile damage than the hanging wall area. The concrete was crushed completely within the fault zone. Thus, the “steel liner + backfilled concrete” structure was inadequate for withstanding the creep deformation of the fault zone.

## Adaptability of multi-layer flexible lining (MFL) tunnel to fault creep

**Fig. 14:**
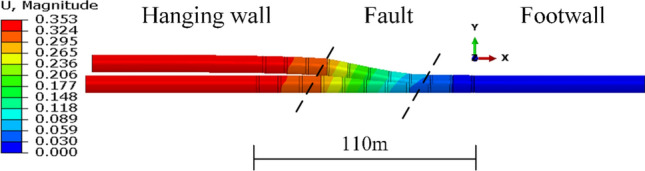
Resultant displacement of steel pipe under fault conditions (m).

According to the calculation results presented above, a lining combination structure was introduced in order to reduce the axial compressive deformation of the steel pipe and minimize the concrete damage, as illustrated in Fig. 2b. Analysis of the results in Fig. 11 revealed that the fault zone had an influence scope of approximately 30 m on each side, so an engineering fortification distance of 110 m was used in the calculation. Figure 14 shows the resultant displacement of the steel pipe, with all the deformation occurring within the engineering fortification zone, indicating that the selection of the fortification distance was adequate. The addition of the bellows joint enhanced the overall flexibility of the steel pipe, resulting in a maximum resultant displacement of 353 mm, which was slightly larger than the resultant displacement of the steel-lined tunnel.

**Fig. 15:**
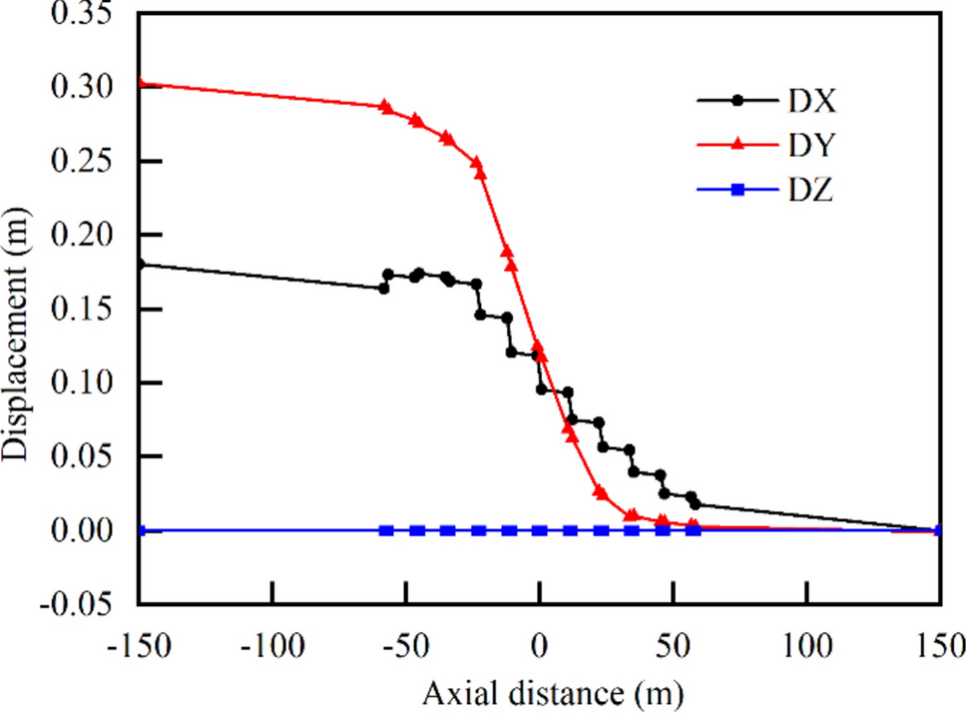
Distribution curves of central displacements of pipeline sections along pipe axis.

The distribution curves of the steel pipe section center displacements along the axis of the pipe, DX–z, DY–z, and DZ–z, are shown in Fig. 15. The displacements in the X- and Y-directions varied significantly due to the fault dislocation, and the two values along the abscissa gradually decreased from left to right. The central displacement of the leftmost pipeline section in the X-direction was approximately 0.17 m, which was equal to the horizontal displacement of the fault. After compensating for the negative displacement caused by gravity, the Y-direction displacement was also roughly 0.3 m, which was equal to the vertical displacement imposed on the surrounding rock. When analyzing the deformation of the bellows joints separately, it was observed that as the fault moved, the deformation of the bellows joints deformed with a roughly linear trend along the Y-direction. The deformation difference between the steel pipes at both ends of the bellows joints in the axial (X-axis) direction was relatively more significant. The axial compressive displacement of the steel pipe was very small, with the majority of the deformation occurring at the bellows joint.

**Fig. 16:**
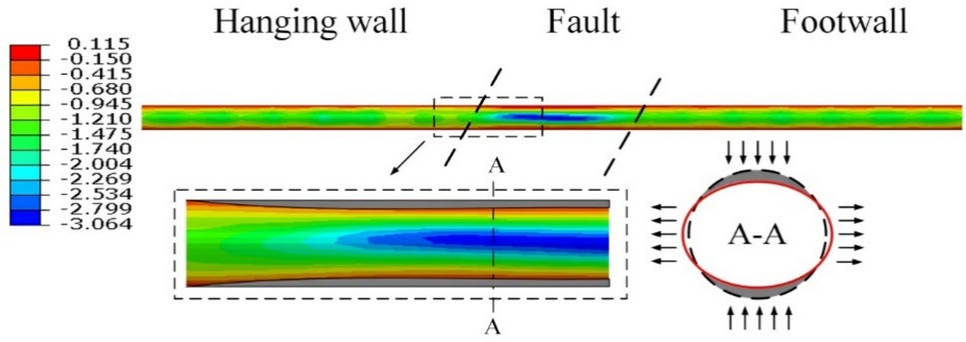
Cushion radial deformation (mm).

Based on the calculation results, a graph of the cushion deformation was generated for a fault displacement of 0.3 m. Figure 16 shows that the magnitude of the cushion deformation ranged from − 3.06 to 0.12 mm, with a maximum compression of 3.06 mm. The compression mainly occurred at the waist position of the cushion in the fault zone, while deformation in other areas was concentrated below 1.5 mm. The calculation model used a continuous cushion outside the steel liner, and the deflection of the pipe sections in the middle of the fault zone caused local tension at the top and bottom of the cushion.

To clarify the role of each part of the lining structure in the normal operation and failure process caused by fault dislocation in the tunnels, in addition to the conventional steel-lined tunnel (Scheme 1) and MFL tunnel (Scheme 5), three additional lining types were introduced based on the steel-lined tunnel (steel liner + concrete) by adding a bellows joint, soft cushion layer, and flexible joint, as shown in Table 5.

**Table 5:** Contents of different lining types.

Scheme	Structural style
S1	Steel liner, concrete
S2	Steel liner, concrete, **bellows joint**
S3	Steel liner, concrete, **cushion layer**
S4	Steel liner, concrete, **flexible joint**
S5	Steel liner, concrete,** bellows joint**, **flexible joint**, **cushion layer**

**Fig. 17:**
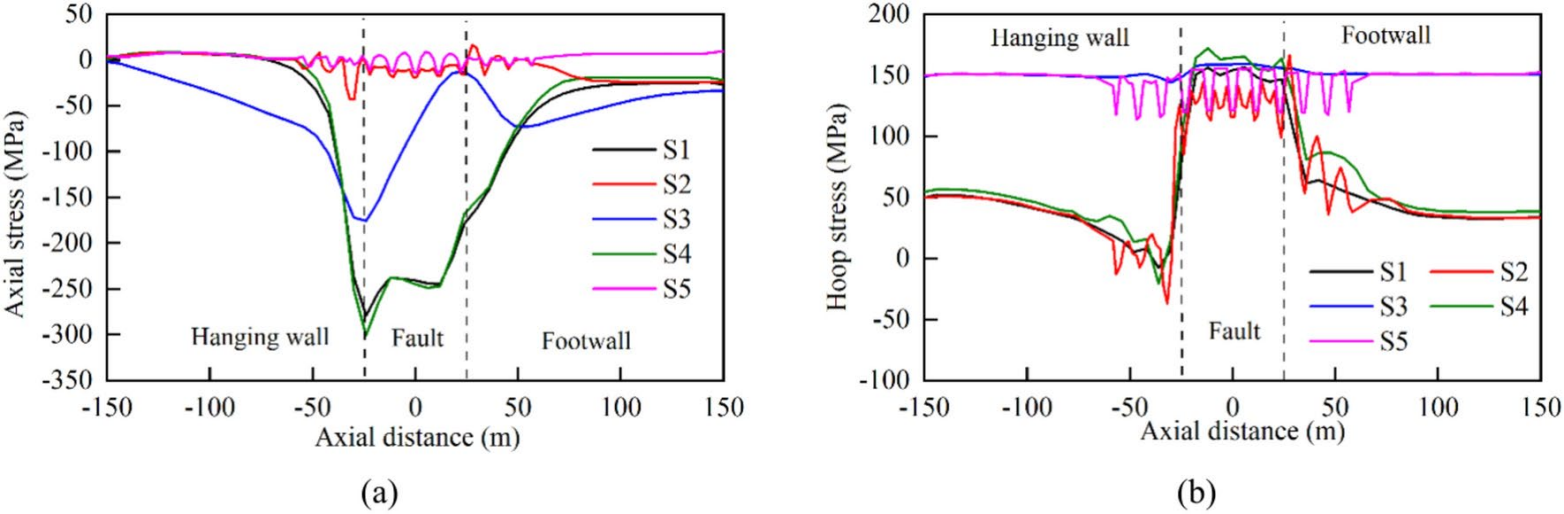
Stress curves of steel pipes with different lining types: (**a**) axial stress and (**b**) hoop stress.

Figure 17 shows the stress variation curves for steel liners with different structural types in the axial and hoop directions. Notably, the addition of a soft cushion layer between the steel liner and concrete resulted in a reduction in the load-carrying capacity of the concrete against the water pressure and a more uniform stress distribution in the steel liner. Specifically, the stress in the steel liner outside the fault influence zone increased significantly, whereas the axial stress peak value inside the zone decreased slightly, and the hoop stress peak value changed less noticeably. The axial stress peak value of the steel liner inside the fault influence zone decreased by 37.4%, from − 280.8 to − 175.8 MPa.

Adding flexible joints into the concrete increased its overall flexibility, leading to greater deformation between the sections and thus increasing the degree of stress concentration in the steel liner. As a result, the addition of flexible joints had little effect on reducing the stress in the steel liner. In contrast, adding bellows joints to the steel liner resulted in a significant improvement in the stress distribution. Compared with the conventional steel-lined scheme, the axial stress peak value of the steel liner decreased by 84.6%, from − 280.8 to − 43.3 MPa.

The compressive and tensile damage volumes of the tunnels were extracted for the different structural types under normal operating and fault dislocation conditions to assess the degree of concrete damage. Based on the extent of the element damage, the element damage was classified into three categories: “0–0.3,” “0.3–0.6,” and “greater than 0.6.” Within these categories, the element damage was further divided into low, medium, and high grades (T_ij_ or C_ij_, i from 1 to 5, j from 1 to 3). The relative compressive and tensile damage ratios (R_ij_) of the concrete were calculated, taking the total volume of concrete within a 110-m fortification distance (V_Total_) as the basis, i.e.,

6 $$R_{ij} { = }\frac{{T_{ij} \, or \, C_{ij} }}{{V_{Total} }},$$

7 $$R_{i1} + R_{i2} + R_{i3} = 1.$$

**Fig. 18:**
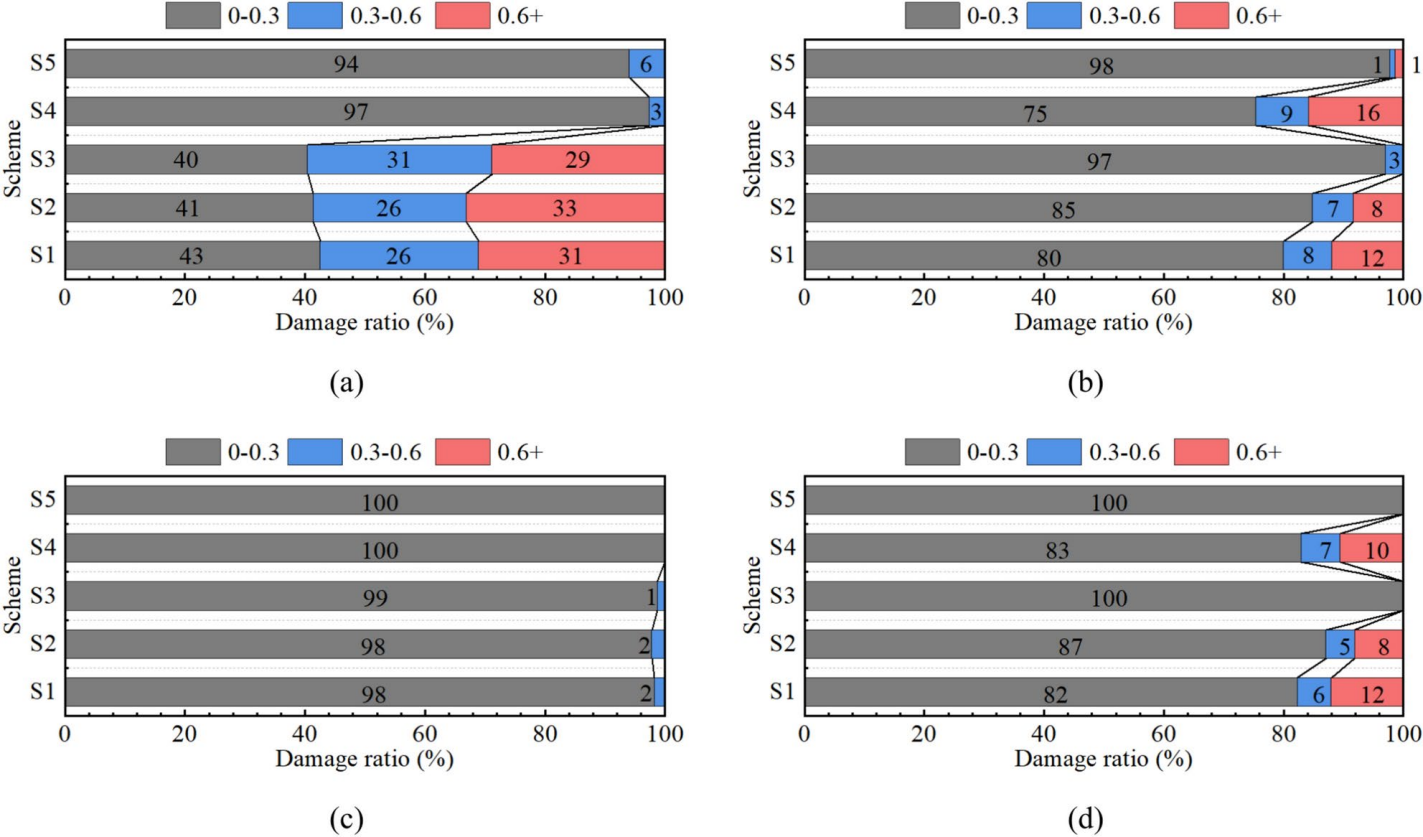
Damage ratio of concrete for different lining types: (**a**) compressive damage under fault dislocation, (**b**) tensile damage under fault dislocation, (**c**) compressive damage under normal operation, and (**d**) tensile damage under normal operation.

The volume ratio of each grade was then determined by the Eq. (6). The calculation results are presented in Fig. 18.

It can be observed that adding bellows joints (S2) or a cushion layer (S3) to the conventional structure did not significantly reduce the concrete compressive damage based on the comparison of the data in Fig. 18a. Additionally, after adding bellows joints to the steel pipes, a higher proportion of elements with a damage level above 0.6 was observed. This was because adding bellows joints caused a loss of radial support inside the concrete at the corresponding position of the joint, making it easier to crush and concentrate the concrete damage. In contrast, schemes S4 and S5 using flexible joints showed a significant reduction in the concrete compressive damage.

The comparison of the data in Fig. 18b showed that adding bellows joints (S2) or flexible joints (S4) to the conventional structure did not significantly reduce the concrete tensile damage. The use of flexible joints even increased the tensile damage volume of the concrete elements. This was because the strength of the structure was reduced after the addition of the flexible connection segment. The concrete segments more easily produced radial tension cracks under internal pressure and fault displacement. As can be seen from the figure, the tensile damage in schemes S3 and S5 was greatly reduced. In the conventional steel-lined scheme, the steel pipe and concrete were in a joint-bearing state. The cushion layer absorbed the expansion displacement of the steel pipe caused by the internal water pressure during normal operation, reducing the outward transmission of the water pressure and consequently reducing the concrete tensile damage significantly.

A horizontal comparison of the data presented in Fig. 18a, b reveals that the backfilled concrete underwent significant deformation during fault displacement. During this process, the concrete experienced both compressive and tensile stresses, which created a complex stress condition, making it challenging to address fault creep through a single structural measure. The axial-direction pressure of the concrete in the fault zone is worth discussing. The axial-direction sections were in hard contact, and the pressure could be directly transmitted, causing the concrete to be crushed in a large area. It is precisely for this reason that the ratio of high-grade damage in schemes S1, S2, and S3 reached a concerning distance of one-third of the total length. By comparing the results in Fig. 18a–d, we found that the compressive damage in all the schemes was mainly concentrated in the low grade under normal operating conditions, and compressive damage mainly occurred during fault dislocation. Conversely, tensile damage mainly occurred during normal operation when the steel pipes were filled with water.

According to the above analysis, the bellows joint mainly absorbed the axial deformation and transverse shear stress of the pipe, which had a good effect in reducing the steel pipe stress. Meanwhile, the soft cushion layer not only reduced the proportion of concrete-bearing water pressure but also absorbed part of the displacement of the concrete segment, making the structural stress more uniform. The flexible joint increased the deformation space between the concrete segments, allowing damage during fault displacement to concentrate on the flexible connection. This created discontinuous surfaces of the axial force and reduced the transmitted axial pressure, thereby avoiding the overall crushing of the concrete. The MFL structure combined the advantages of various structural measures and to some extent overcame their respective shortcomings, showing excellent performances in reducing the steel pipe stress and minimizing concrete compressive and tensile damage.

## Analysis for optimization of structural design parameters

The analysis presented in this section was based on the previous numerical modeling approach and structural arrangement. The effects of factors on the internal force response and fracture resistance of the tunnel structure were analyzed. These factors include the length of the concrete segment, the thickness of the cushion, and the number of bellows joints.

### Effect of concrete segment length

This section investigates the impact of the concrete segment length on the structural behavior. In light of the common practice in tunnel construction, where concrete lining trolleys usually range from 6 to 12 m^9,19^, concrete segment lengths of 6, 8, and 10 m were employed. While keeping the other parameters of the lining combination scheme constant, the concrete segment length was varied. The von Mises stress of the steel pipe and the deformation of the bellows joint showed negligible changes as the length of the segment increased from 6 to 10 m. Instead, the change mainly occurred in the concrete.

**Fig. 19:**
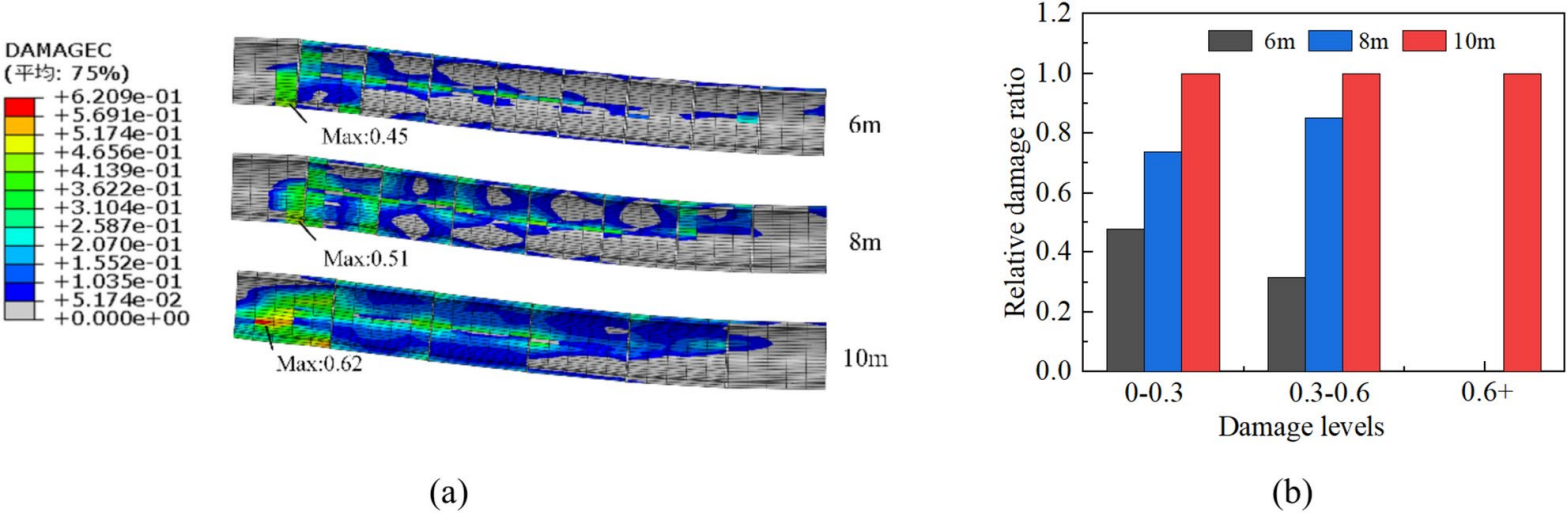
Compressive damage of concrete for three length schemes: (**a**) damage contour and (**b**) relative damage ratio based on 10-m scheme.

The analysis showed that the tensile damage of the concrete, which was predominantly caused by the water pressure, remained similar in the schemes with the three lengths. However, Fig. 19a shows that the maximum compressive damage values occurred at the bottom of the concrete tube in all the schemes, which increased with the increase in the lining segment length. Shortening the concrete segment length could simultaneously reduce the damage scope and extent. The established damage division intervals were employed to calculate the relative damage ratio by comparing the volume of the compressed damage elements in each interval, taking the segment length of 10 m as the reference. To gain further insight into the impact of the segment length, the relative damage ratio is presented in Fig. 19b.

Based on the results in Fig. 19b, it is clear that reducing the length of the concrete segments is an effective method for mitigating compressive damage in tunnels. Compared to the 10-m calculation scheme, the 6- and 8-m segments exhibited 50% and 20% less damage, respectively, in the 0–0.3 interval. Moreover, the 6-m scheme achieved a damage reduction of up to 70% in the 0.3–0.6 level, and there were no damaged elements in the greater-than-0.6 interval for both the 6- and 8-m schemes.

**Fig. 20:**
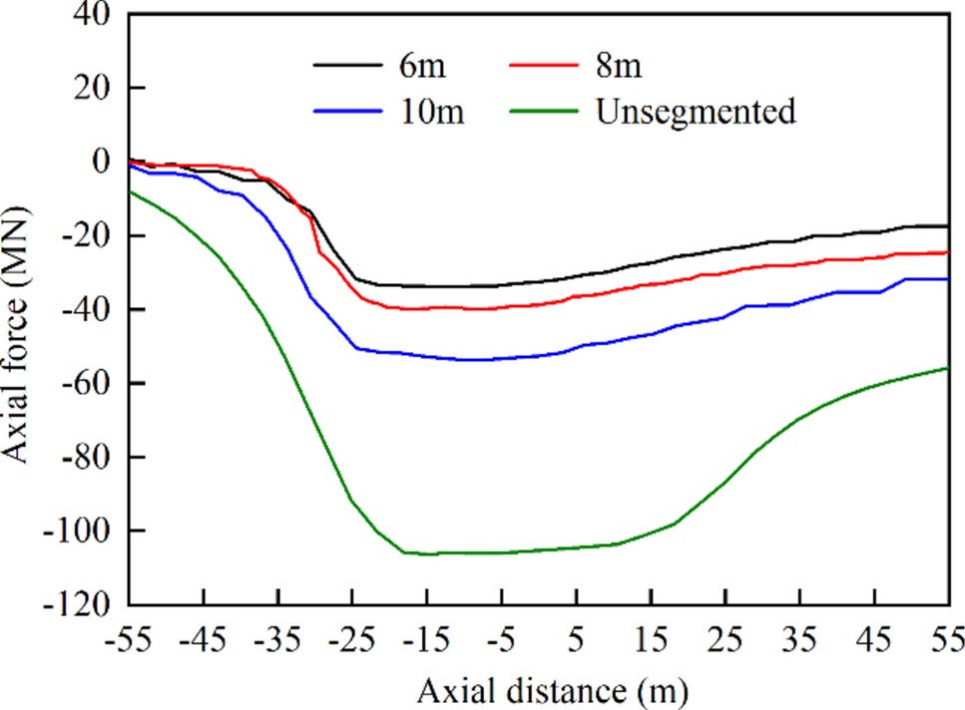
Axial forces inside concrete for different schemes.

Based on the analysis of the axial force within the concrete (in Fig. 20) and a comparison with the unsegmented scheme, it is apparent that the inclusion of flexible joint segments could greatly diminish the axial force along the concrete. Further reduction of the concrete length could lead to even smaller axial forces. However, the use of shorter concrete segments could result in increased construction difficulties and project costs, which should be carefully considered in the design process.

### Effect of flexible joint length

**Fig. 21:**
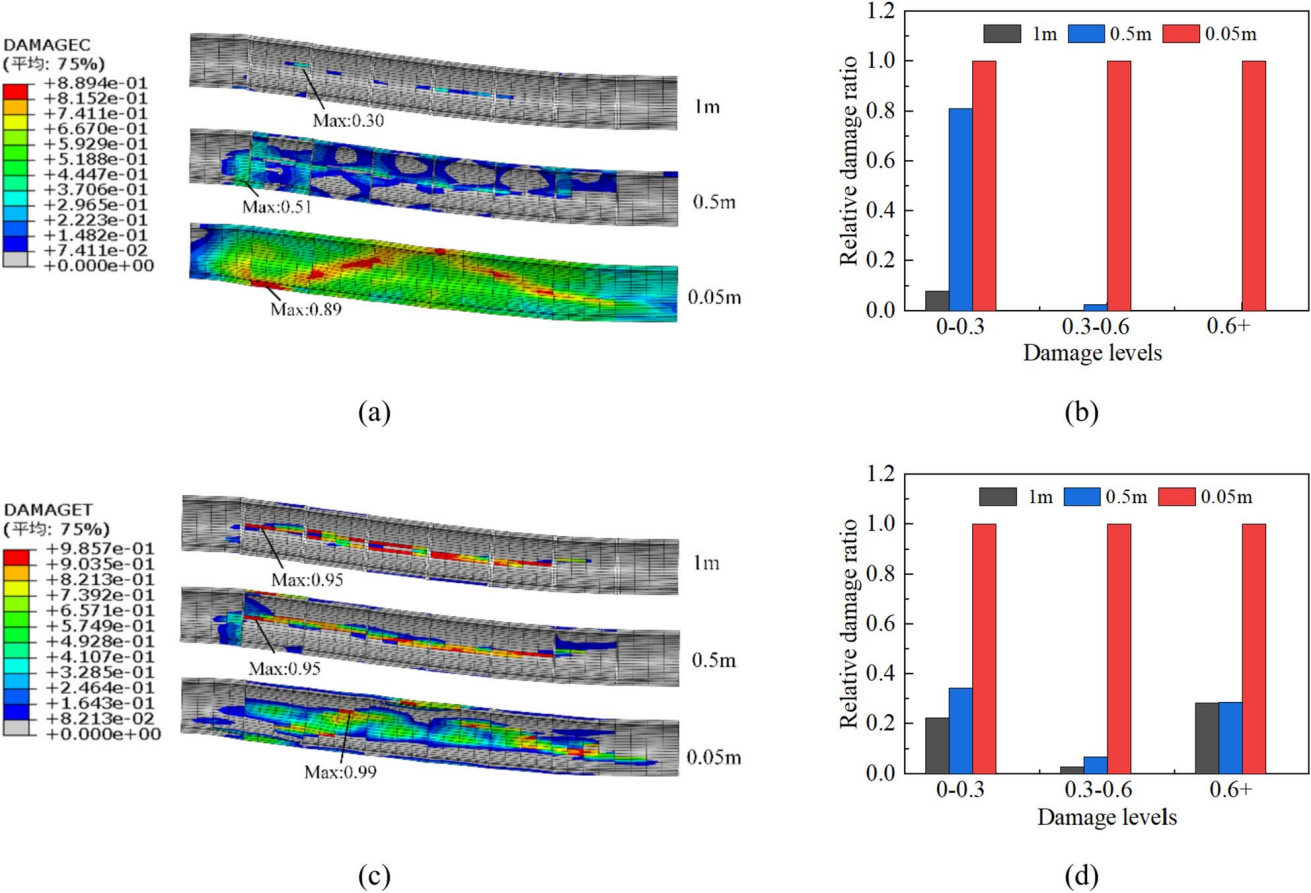
Compressive and tensile damage of three flexible joint lengths: (**a**) compressive damage contours, (**b**) compressive relative damage ratio based on 0.05-m scheme, (**c**) tensile damage contour, and (**d**) tensile relative damage ratio based on 0.05-m scheme.

Flexible joints often experience damage before the concrete segments due to their low moduli and large amounts of deformation. They can effectively absorb the deformation caused by the fault and protect the concrete sections from failure. To investigate the effect of the flexible joint length on the tunnel’s structural response, three commonly used lengths of 0.05, 0.5, and 1 m were adopted in this study, while keeping the other numerical parameters of the lining combination scheme constant. As the length of the flexible joint increased from 0.05 to 1 m, the von Mises stress of the steel pipe and the deformation of the bellows joints underwent little change, and the change mainly occurred in the concrete. The concrete compressive damage and its corresponding relative damage ratio are shown in Fig. 21a, b, respectively. The tensile damage of concrete and its corresponding relative damage ratio are shown in Fig. 21c, d, respectively.

When using flexible joints with a length of 0.05 m, typical diagonal shear cracks appeared in the concrete within the fracture zone. Additionally, the tensile damage area showed a wide belt-like distribution. Meanwhile, as the length of the flexible joint increased to 0.5 m, the compressive damage zone showed a discontinuous block-like distribution, while the area of tensile damage showed a relatively narrow stripe shape. As the length of the flexible joint continued to increase, the degree of damage decreased further.

In the 0.5-m scheme, most of the compressive element damage values were concentrated in the 0–0.3 interval range, and only a few sporadic compressive areas existed at the waist of the concrete pipe in the 1-m scheme. The distribution and level of concrete tensile damage were approximately the same in the 0.5- and 1-m schemes. As shown in Fig. 21b, d, increasing the length of the flexible joint sharply decreased the concrete damage in tension and compression. It is important to note that a too long of a flexible joint may reduce the effectiveness of the concrete lining support.

### Effect of cushion thickness

**Fig. 22:**
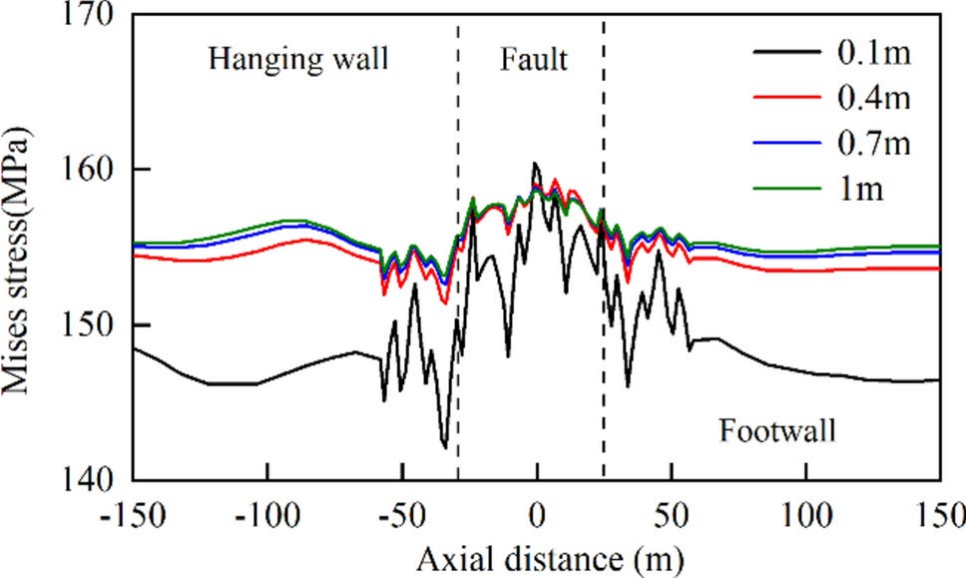
Von Mises stress curves for steel pipes with different cushion thicknesses.

In the MFL structure, the cushion layer plays a crucial role. To investigate the effect of the cushion layer thickness on the stress and deformation of the lining structure, the cushion layer thickness was assumed to be 0.1, 0.4, 0.7, and 1 m. As the thickness of the cushion layer was increased from 0.1 to 1 m, the range of damage caused by compressive and tensile stresses on the concrete gradually expanded, while the degree of damage gradually decreased. The changes in both were not particularly significant. Furthermore, the thicker the cushion layer was, the lower the water pressure transmitted outward from the steel pipe to the concrete became. The von Mises stress of the steel pipe outside the fault range increased sharply, and the maximum stress of the steel pipe within the fault range decreased slightly when the cushion thickness increased from 0.1 to 0.4 m (Fig. 22). However, as the cushion thickness continued to increase, the rate of increase of the von Mises stress gradually decreased.

**Fig. 23:**
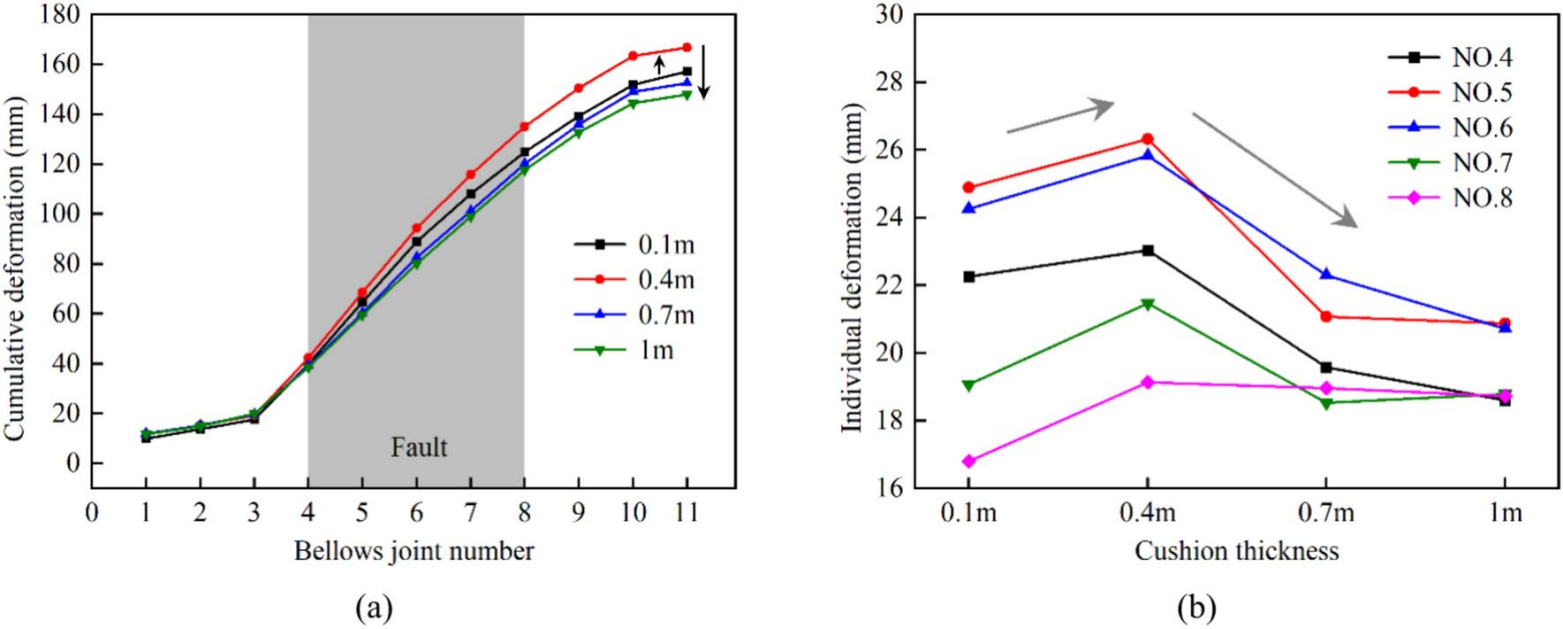
Relationship between bellows joint deformation and cushion thickness: (**a**) total cumulative deformation of all bellows joints and (**b**) individual deformation of bellows joints within fault zone under different cushion thicknesses.

To investigate the relationship between the deformation of the bellows joints and the cushion thickness, we assigned numbers 1–11 to the bellows joints based on their order from the hanging wall to the footwall. The effect of the cushion thickness on the deformation of the bellows joints is shown in Fig. 23.

**Fig. 24:**
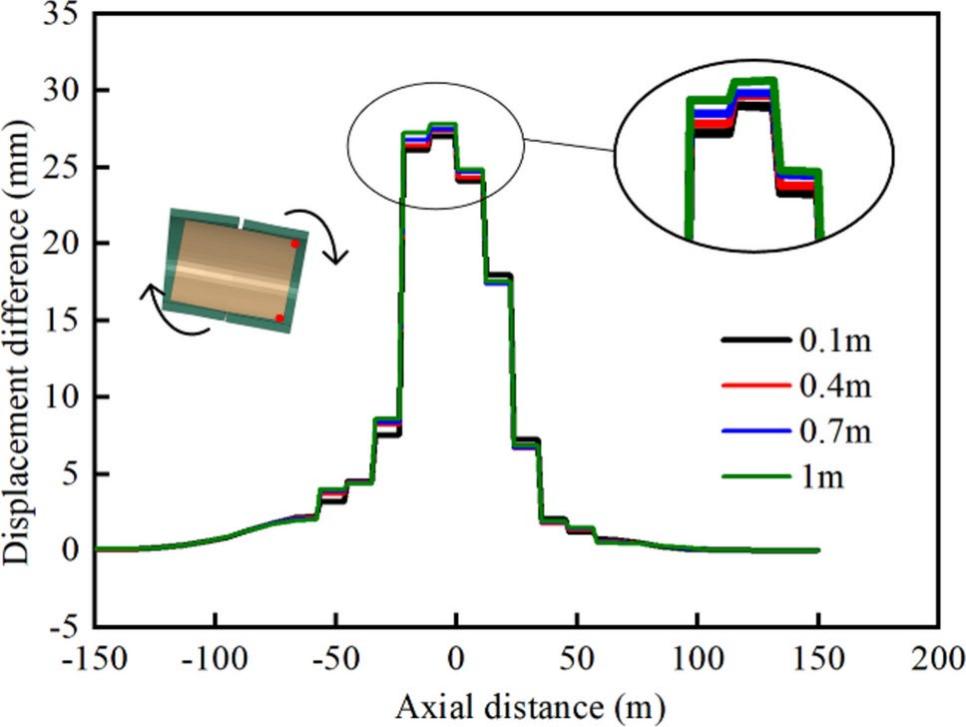
Axial displacement difference curves of top and bottom points of pipe cross section.

As per conventional understanding, a thicker cushion layer allows for more space for the bellows joint, which can increase the overall flexibility of the steel liner. This makes the structure more susceptible to deformation, resulting in greater total cumulative deformation. It is interesting that with the increase in the cushion thickness, the total cumulative deformation and individual deformation of the bellows joints showed a trend of increasing before decreasing. To explain this phenomenon, the axial displacement difference between the top and bottom points of the cross sections at both ends of each pipe segment were extracted, as shown in Fig. 24. This value reflected the degree of deflection of the steel segment. It can be seen that the rotation of the pipe segments was more pronounced near the middle of the fault zone.

The cushion layer in the MFL structure served dual purposes: on the one hand, it reduced the deformation transmitted from the concrete to the steel pipes by providing a buffering effect; on the other hand, it created the deformation space for the bellows joints and the steel pipe, thereby regulating the overall flexibility of the structure. Initially increasing the cushion layer thickness allowed for greater deformation of the bellows joints, resulting in an increase in the total cumulative deformation of the joints. This suggested that within a reasonable range, increasing the thickness of the cushion layer could better facilitate the displacement compensation function of the bellows joints. At this point, the second purpose of the cushion layer became dominant. Subsequently, as the cushion layer thickness continued to increase, the cushion partially absorbed displacement of the concrete, thereby decreasing the transmission of displacement from the concrete to the steel pipe. At this stage, the first purpose of the cushion layer became dominant. Additionally, the rotation of the steel pipe also contributed to the absorption of the displacement caused by the fault, leading to reduced compensation at the bellows joints.

In terms of the anti-fracture design of the lining structures, using an overly thick cushion layer is beneficial for minimizing the extent of the concrete damage. Nevertheless, such an approach may augment the von Mises stress of the steel pipe and diminish the displacement compensation function of the bellows joint. The thicker the cushion layer is, the larger the tunnel diameter is, and the less economical the project becomes. Consequently, the ideal thickness of the cushion layer should be ascertained via precise calculations.

### Effect of number of bellows joints

**Fig. 25:**
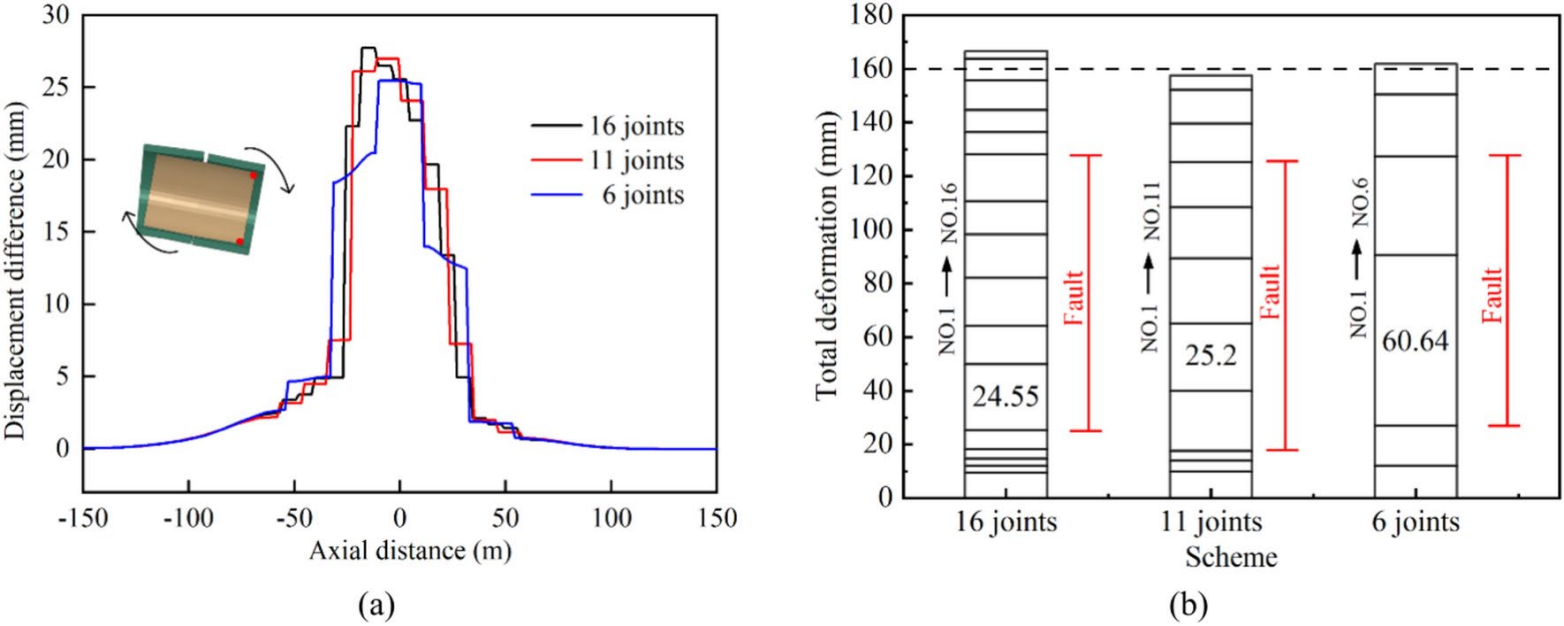
(**a**) Axial displacement difference curves of top and bottom points of pipe cross section and (**b**) relationship between bellows joint deformation and number of bellows joints.

The extrusion deformation of a steel pipe is mainly absorbed by bellows joints, and the number of bellows joints will directly affect the adaptability of the MFL structure to fault dislocations. With the engineering fortification distance and other parameters of the numerical model of the combination scheme unchanged, the number of bellows joints was varied by adjusting the lengths of the steel pipe segments. The number of bellows joints was set to six, 11, and 16. Increasing the number of bellows joints resulted in shorter lengths of the steel pipe segments, making the overall structure more flexible, as shown in Fig. 25a. This makes it easier for the steel lining to deflect in the direction of the fault dislocation, thereby reducing the damage caused by the extrusion of the steel pipe itself and the concrete. The effect of changing the number of bellows joints on the concrete was limited, with the main impact being on the stresses of the steel pipes and the deformation of the bellows joints.

In contrast to the stress level of 184.8 MPa observed when using only six bellows joints, the use of shortened-steel pipe segments with 16 bellows joints resulted in a lower maximum von Mises stress of 154.7 MPa, representing a reduction of 16.3%. The total cumulative deformation values of all the bellows joints for different numbers of bellows joints are plotted in Fig. 25b.

The total deformation of the bellows joints remained near 160 mm with a small variation range; in all three cases, the bellows joints with the largest deformation were located at the intersection of the hanging wall with the fault zone, and the maximum deformation of a single bellows joint reached 60.64 mm when the number of bellows joints was six, while the value was 24.55 mm when the number of bellows joints was 16, which was 2.47 times greater than the value with six bellows joints. However, the value was 25.2 mm when the number of bellows joints was 11, which was not significantly different from the scheme with 16 joints.

From the perspective of the anti-fault design of the lining structure, using more bellows joints was beneficial for improving the ability of the tunnel to resist fault displacement. However, there was a marginal diminishing effect when increasing the number of bellows joints. That is, after reaching a certain number, further increasing the number of bellows joints had limited effects. The deformation differences between each bellows joint were also significant. Bellows joints with noticeable deformation were predominantly concentrated within the fault zone. When there were too many bellows joints, the deformation of the bellows joints near the edge of the engineering fortification zone was very small and did not have a corresponding effect. Therefore, excessive use of bellows joints in engineering projects can substantially increase the project costs. However, when there are too few bellows joints, deformation is likely to concentrate on a small number of such joints, which can cause structural breakdown and loss of normal operating capacity.

## Discussion

**Fig. 26:**
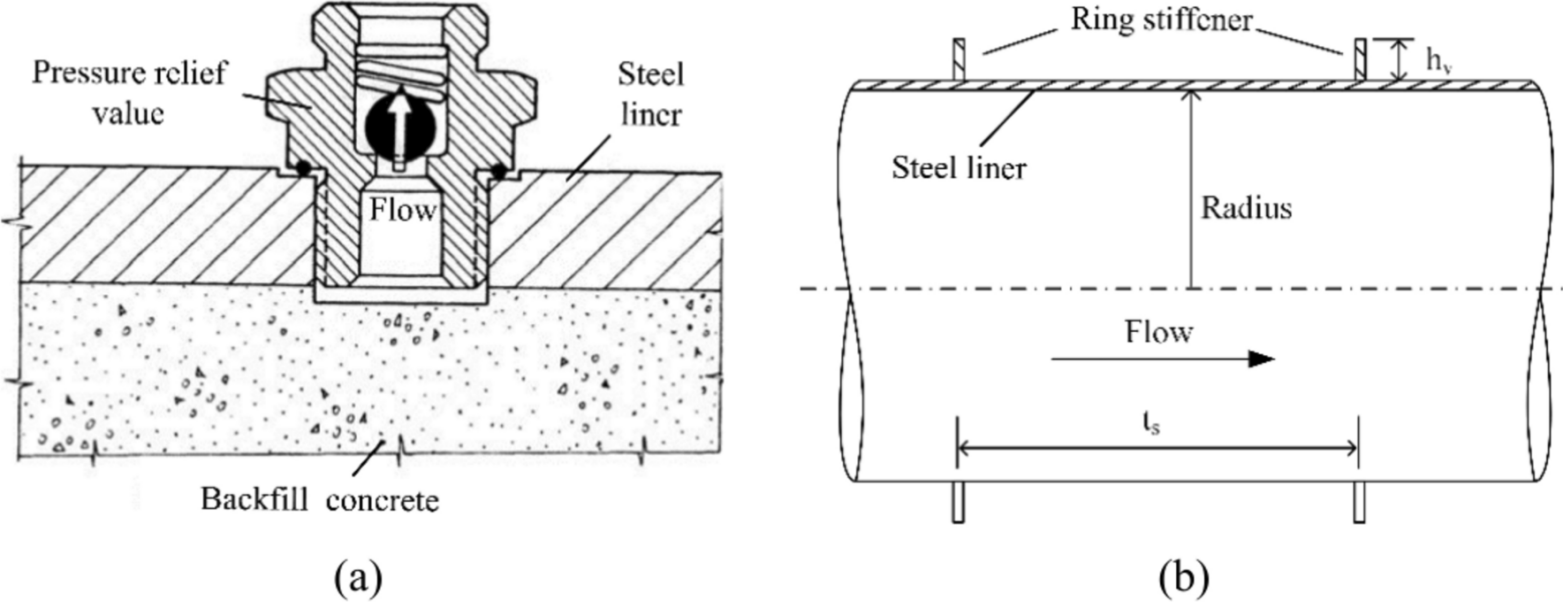
Structures for improving stability of steel pipe against external pressure: (**a**) pressure relief value and (**b**) ring stiffener.

This article proposes an innovative structure that can effectively enhance the robustness of high-pressure water transmission tunnels crossing faults against creep deformation, enabling long-term stable operation of an engineering project. However, there is a corresponding limitation: it is more suitable for tunnels with low external water pressures. When the tunnel is under maintenance, the internal pressure of the pipeline is zero, and the external water pressure acts entirely on the steel liner. Due to the existence of a cushion layer, flexible joints, and other structures, the steel pipe may face a risk of local instability. Whether the structure can meet the stability requirements for external pressure resistance needs to be verified in detail. When the structure does not meet the requirements for external pressure resistance, measures such as increasing the thickness of the pipe wall, setting ring stiffeners, and installing pressure relief valves can be adopted to improve the pipe’s external pressure resistance. Some structures are shown in Fig. 26. Previous scholars^35–38^ have conducted extensive research on these methods, proving their feasibility and reliability. However, it remains to be studied how the adaptation of the structure to fault dislocations will be affected after implementing these measures.

## Conclusions

Based on the results, the following conclusions can be made:

1. An MFL structure suitable for high-pressure water transmission tunnels crossing active faults was proposed. The research confirmed the rationality and feasibility of this new tunnel lining combination structure, providing a feasible direction for the structural design of high-pressure water transmission tunnels to cope with fault creep deformation.
2. The numerical calculations showed that if the structure had shorter concrete segments or longer flexible joints, it could better cope with the compressive and shear effects of the fault on the structure and reduce the damage range of the concrete.
3. When the cushion thickness ranged between 0.1 and 0.4 m, a thicker cushion layer could facilitate the deformation of the bellows joints, thereby improving their capacity to accommodate fault displacement. Increasing the cushion thickness enabled the individual steel pipe sections to accommodate fault displacement more effectively through rotational movements. Nevertheless, when the cushion thickness increased beyond this range, the benefits became less pronounced and could lead to enlargement of the tunnel diameter. Therefore, it is recommended to select an appropriate cushion layer thickness that depends on the fault displacement in the engineering design.
4. The use of an appropriate number of bellows joints can enhance the ability of a tunnel to withstand fault displacement. Overuse or underuse of bellows joints can impact project costs and structural stability and therefore must be balanced within an acceptable range. Additionally, bellows joints located at the edge of the fault zone are susceptible to failure and require special attention.

## Data Availability

The datasets used and/or analyzed during the current study available from the corresponding author on reasonable request. Competing interests The authors declare no competing interests.
